# Loss of HCN2 leads to delayed gastrointestinal motility and reduced energy intake in mice

**DOI:** 10.1371/journal.pone.0193012

**Published:** 2018-02-21

**Authors:** Daniel W. Fisher, Phillip Luu, Neha Agarwal, Jonathan E. Kurz, Dane M. Chetkovich

**Affiliations:** 1 Davee Department of Neurology and Clinical Neurosciences, Northwestern University Feinberg School of Medicine, Chicago, IL, United States of America; 2 Department of Pediatrics, Northwestern University Feinberg School of Medicine, Chicago, IL, United States of America; 3 Department of Neurology, Vanderbilt University Medical Center, Nashville, TN, United States of America; University of Texas Medical Branch, UNITED STATES

## Abstract

Hyperpolarization-activated Cyclic Nucleotide-gated (HCN) channels are important regulators of excitability in neural, cardiac, and other pacemaking cells, which are often altered in disease. In mice, loss of HCN2 leads to cardiac dysrhythmias, persistent spike-wave discharges similar to those seen in absence epilepsy, ataxia, tremor, reduced neuropathic and inflammatory pain, antidepressant-like behavior, infertility, and severely restricted growth. While many of these phenotypes have tissue-specific mechanisms, the cause of restricted growth in HCN2 knockout animals remains unknown. Here, we characterize a novel, 3kb insertion mutation of *Hcn2* in the Tremor and Reduced Lifespan 2 (TRLS/2J) mouse that leads to complete loss of HCN2 protein, and we show that this mutation causes many phenotypes similar to other mice lacking HCN2 expression. We then demonstrate that while TRLS/2J mice have low blood glucose levels and impaired growth, dysfunction in hormonal secretion from the pancreas, pituitary, and thyroid are unlikely to lead to this phenotype. Instead, we find that homozygous TRLS/2J mice have abnormal gastrointestinal function that is characterized by less food consumption and delayed gastrointestinal transit as compared to wildtype mice. In summary, a novel mutation in HCN2 likely leads to impaired GI motility, causing the severe growth restriction seen in mice with mutations that eliminate HCN2 expression.

## Introduction

Hyperpolarization-activated Cyclic Nucleotide-gated (HCN) channels are primarily expressed in neural and cardiac tissues where they influence the excitability of both pacemaking and non-pacemaking cells by conducting a depolarizing, hyperpolarization-activated inward current (I_h_)[1, 2]. HCN channels form both hetero- and homo-tetramers in a “dimer-of-dimers” configuration from four pore-forming subunits, HCN1-4[1, 2]. Differing expression patterns, channel gating kinetics, interaction with accessory subunits[2–4], and responsivity to modulators such as cyclic nucleotides distinguish the effects of each subunit on cellular and somatic processes[1, 2].

HCN2 is expressed at high levels in both the heart and nervous system, and many of the phenotypes that result from HCN2 deletion reflect these expression patterns. Though homozygous HCN2-KO mice are born in Mendelian ratios from heterozygous parents, they begin to display a severe phenotype between the second and third weeks of life[5, 6]. Most notably, HCN2-KO mice display cardiac dysrhythmias, frequent epileptiform spike-wave discharges (SWDs), ataxia, tremor, reduced sensitivity to neuropathic and inflammatory pain, antidepressant-like behavior, infertility, and severely restricted growth[5–8]. Of these numerous phenotypes, several have been characterized on the cellular level, but the severe growth defect has yet to be recapitulated with cardiac or neuron-specific *Hcn2* deletion [5–8] [9] [6, 10]. Here, we present the first detailed characterization of a spontaneous mutation in *Hcn2* found in Tremor and Reduced Lifespan 2 (TRLS/2J) mice. This insertion mutation causes complete loss of HCN2 protein expression and a phenotype consistent with other mice lacking HCN2 expression. In addition, we investigated the growth defect in TRLS/2J mice to determine a mechanism causing this phenotype.

## Materials and methods

### Animals

TRLS/2J mice arose spontaneously in a breeding colony of WB/ReJ-*Kit*^*W*^/J mice as being weak and of small size with early mortality. These mice were then backcrossed to C57BL/6J mice for 4 generations without seeing signs of the Kit^W^ allele, indicated by a white ventral spot on a black fur coat. TRLS/2J heterozygous mice (*Trls2*^*+*/-^) were set up to breed at Northwestern University, resulting in wildtype (*Trls2*^+/+^), *Trls2*^+/-^, and homozygous TRLS/2J (*Trls2*^*-/-*^) mice at the expected Mendelian ratios. Similar to other mice lacking HCN2 expression, both male and female *Trls2*^*-/-*^ mice were infertile. After sequencing *Hcn2* in the TRLS/2J mice, primers were developed for genotyping as follows: WT-F-5’ GATGTTGCCTCACAGAGAATGC, Common-R-3’ CCTCCCTTTGACTCTCTCCTCA, and MUT-F-5’ GTTCTTTACTGGCATCGGCTACG. The wildtype allele created a 473 bp band and the mutant allele created a 289 bp band. Upon weaning, TRLS/2J mice’s diet of Teklad LM-485 Mouse/Rat Diet 7912 (Envigo; Madison, WI) was supplemented with Recovery DietGel (ClearH20; Westbrook, Me), which was changed at least twice weekly. Otherwise, mice had access to food and water *ad libitum*. Male and female mice were used for all studies, and mice were age-matched. Mice were maintained on a 14:10 hour light:dark cycle. Animals were euthanized by inhalation with carbon dioxide or isoflurane followed by cervical dislocation or decapitation. All animal experiments were performed according to protocols approved by the Institutional Animal Care and Use Committee of Northwestern University (IS00001380).

### HCN2 gene sequencing

Sanger sequencing was performed by Northwestern’s Center for Genetic Medicine (CGM) using PCR products generated by lab-designed primers (See Table 1). PCR products were purified with QIAquick Gel Extraction Kits (Qiagen; Germantown, MD) before being sent to CGM for sequencing. Sequence chromatograms were verified for quality using A Plasmid Editor (aPE; University of Utah; Salt Lake City, UT) and BLAST (NCBI; Bethesda, MD) was used to map sequencing reads to canonical sequences of the mammalian genome.

**Table 1:** Primers used to sequence *Hcn2*.

**HCN2 Exon Primers**	
**Primer**	**Sequence**
1F	GCTTGCTGGCCAGAGCCTCAGTT
2F	AAGGCCAAGAGCTGTGAGCA
3F	TGGGACTCTCATGGTCACACG
5F	GTGGCATTAGGGTGGACCTG
4F	GATGTTGCCTCACAGAGAATGC
6-7F	GAGTTACCAAGCCAGTCACTGAG
8F	CATGGCTTTGGTGTGGATGACG
1R	CCGAGGTCACCATCCGGGACGT
2R	ATGCTTGGTCCTCTCCCTGC
3R	GGCACAAGGGCAAATGGGAG
4R	CCTCCCTTTGACTCTCTCCTCA
5R	GGCTAAAACTCCCCAAGTCCTGTG
6-7R	ATGCCATGCCATGCCTCTACTT
8R	CGTCATCCACACCAAAGCCATG
**Insertion Mutation Primers**	
**Primer**	**Sequence**
3kb-1 F	GTTCTTTACTGGCATCGGCTACG
3kb-2 F	CCTTCCGGTCTTGCTCATG
3kb-2 R	CTCTGGTGTGTGAAATTTCAGAGG
3kb-3 F	CAGCTCCTCTATGTACTTGCTCTC
3kb-3 R	GGACTTGGTAATGTGTAAGGGTCAC
3kb-4 F	AAGGGACTTGACACGGATACAAC
3kb-4 R	AGCATATGTAAAGACTGTGGGACTT

### Western blot

Western blotting was performed as previously described[11]. Samples were homogenized in RIPA buffer and then sonicated briefly on ice. Sample were then centrifuged at 21000g for 10min, supernatant was collected, and sample buffer containing β-mercaptoethanol was added. Samples were resolved on a 10% SDS-PAGE gel and transferred to PVDF. All blots were blocked with 5% Milk in TBS with Tween-20 (0.1%) before adding primary antibodies in blocking buffer at 4C overnight. Primary C-terminal anti-HCN2 antibody used was custom[11] while the N-terminal anti-HCN2 antibody was purchased from Alomone (Jeruselum, Israel). The C-terminal antibody was used at 1:1000 while the N-terminal antibody was used at 1:250. Band intensities were imaged using Li-Cor Odyssey FC Imaging System and ImageStudio software (Lincoln, NE).

### Growth curve

TRLS/2J mice had their weight and length measured starting at postnatal day 7 (P7). Weight was assessed using a portable scale with accuracy to one decimal place in grams, while length was measured with electronic calipers. Mice were weighed once daily until P21, where they were weaned into separate cages by sex and were then weighed every 7 days until P56. Mice used in growth studies were not used in any subsequent studies to avoid the effects of stress associated with these measurements.

### Behavioral testing

For all behavioral experiments, mice were acclimated to the testing room for 30 minutes in singly housed cages, and all tests were performed during the light phase of the dark/light cycle (6:00a to 8:00p). The testing room was sound-proofed, and between each trial of testing, all apparatuses were cleaned with 70% ethanol. The experimenter was blind to the genotypes of the mice to prevent bias.

### Tail Suspension Test (TST)

Antidepressant-like behavior was assessed by the TST, as previously described[7]. Briefly, mice were suspended by their tails for 6 minutes. Immobility time, defined as time spent without movement of the fore or hind limbs, was measured by an observer blinded to genotype.

### RotaRod

The RotaRod assay was performed as previously described[6]. Briefly, the assay took place over 4 trials: two training trials and two test trials. During each trial, the mouse was placed on a rotating rod (Ugo Basile; Varese, Italy) accelerated from 4 to 40 rpm over a 5 min period, and the latency to fall off the rod was recorded. If the mouse did not fall within 5 min, the mouse was removed from the rod and given a latency to fall value of 300s. The two test trials were averaged together to give the final endpoint in this assay.

### EEG

EEG was performed as previously described[12] with the following modifications. Briefly, mice were anesthetized with isoflurane and placed in a stereotaxic frame. A prefabricated mouse headmount (Pinnacle Technologies; Lawrence, KS) was fastened onto the skull with super glue, and 4 small holes were created with a dental drill to allow placement of 4 screw electrodes (2 screws placed 1mm anterior to bregma and 2 screws placed 7mm posterior to bregma, each being 1.5mm lateral to the central sulcus). Dental acrylic was fashioned over the skull to add stability to the headmount, and the scalp was closed with sutures. Mice were allowed 7 days to recover and then were placed in the recording chambers for continuous, 24h, video-EEG recordings, collected with Sirenia Acquisition Software (Pinnacle) and band pass filtered at 1-25Hz. EEG/video analysis was performed offline using MATLAB/EEGLAB (Mathworks; Natick, MA) by manually scrolling through 10 s epochs of EEG data to detect epileptiform activity by an experienced observer blinded to each subject’s genotype. Spike-wave discharges were identified as periods of the EEG exhibiting a distinct 3-8Hz spike-wave morphology, with amplitudes at least twice that of the baseline[10].

### Tail vein blood and serum collection

Tail vein blood was collected in CB 300 Z microvette tubes (Nümbrecht, Germany) at two different time points, one hour after lights on and one hour after lights off. Collected tail vein blood was immediately put on ice and spun down at 4000rpm in an Eppendorf 5424 R Centrifuge (Hauppauge, NY), and the serum was collected and stored at -80C until being assayed.

### Blood glucose measurements

A drop of tail vein blood was applied to a Freestyle Precision Neo glucose meter (Abbott; Alameda, CA) before each tail vein blood draw and again after the blood draw was completed. These two measurements were averaged together for each animal and represented the blood glucose concentration at that time point.

### Pancreatic, pituitary, and thyroid hormone measurements

All hormone concentrations were assessed from serum collected at the one hour post-lights on time point. All hormone assessments were done with kits per the manufacturers’ instructions. The following kits were used: Ultra Sensitive Mouse Insulin ELISA (Crystal Chem; Downer’s Grove, IL), Mouse Pituitary Magnetic Bead Panel (EMD Millipore; Billerica, MA), and Rat Thyroid Hormone Magnetic Bead Panel (EMD Millipore). The insulin ELISA was read on a Spectra Max 190/plus 384 (Molecular Devices Corporation; Sunnyvale, California) and the pituitary and thyroid panels were read with a Luminex 200 (Northbrook, IL). All ELISA assays were run in duplicate for each serum sample, and technical replicates were averaged together to give the final endpoint per mouse.

### Hemotoxylin and Eosin (H&E) staining and microscopy

Mice were euthanized and tissue was immediately collected, fixed in 10% formalin, and sent to the Northwestern Mouse and Histology Phenotyping Laboratory for processing and staining. Sections were cut to 4 μm thickness, mounted on slides, and stained with H&E. Pictures were obtained with a Zeiss Axioplan 2 and AxioVision Software (Jena, Germany).

### Feeding studies

Feeding studies were administered over three weeks, with a different diet provided during each week of testing. Each feeding trial took place over 72hrs, and was broken up into three 24hr periods. *Trls2*^+/+^, *Trls2*^+/-^, and *Trls2*^-/-^ cage mates at 8–10 weeks of age were separated into single housing with the normal diet of rodent chow and diet gel (as described above) removed but water provided *ad libitum*. During the first feeding trial, each mouse was given ~20g of diet gel on the floor of the cage, and the weight of the gel was measured and recorded at the beginning of the 24hr period. At the end of each 24hr period, the amount of remaining diet gel was weighed and recorded, and new diet gel was again weighed, recorded, and put in the mouse’s cage for the next 24hr period. After three 24hr periods (72hrs), the mice were replaced into their original, group housing cages with their original diets of rodent chow and diet gel until the next testing period. The protocol for these trials were repeated each week, with moist and ground chow given during the second feeding trial and diet gel and solid rodent chow both given during the third and final trial (“choice” trial). The average amount of food eaten during each 24hr period was recorded and each 24hr value was averaged together to yield the average amount eaten per 24hr period for each mouse. The average weight of diet gel or hard chow eaten was converted into kcal consumed based on the macronutrient information provided by the vendor (diet gel = 1.2kcal/g; hard chow = 3.1kcal/g).

### Gastrointestinal Transit Time (GTT) assay

Each mouse was 3–4 months old and singly housed 18 hrs prior to each trial in cages without food but with water *ad libitum*, thus acclimatizing the mice to the new environment. At the start of each GTT trial, mice were gavaged with a methylcellulose gel, consisting of 1.5% sodium carboxymethyl cellulose (Sigma-Aldrich; St. Louis, MO) in water mixed with 6% carmine red (Sigma-Aldrich; St. Louis, MO). Immediately after each gavage period, the GTT time started, and an investigator blind to genotype inspected each cage every 5min for presence of red feces, at which point they recorded the time. GTT was defined as the length of time it took for red feces to appear following gavage.

### Statistics

All statistical calculations were performed using GraphPad 6. For comparisons of three groups with one factors, a One-Way ANOVA was used. For comparisons of three groups over time (within trial comparisons), a Repeated Measures ANOVA was used. Tukey’s *post-hoc* test was used for pairwise comparisons following significant ANOVA results. Significance was denoted with an asterisk, representing a p-value < 0.05, and all values were reported as mean±S.E.M.

## Results

### A 3kb insertion mutation leads to loss of HCN2 protein in TRLS/2J mice

**Fig 1:**
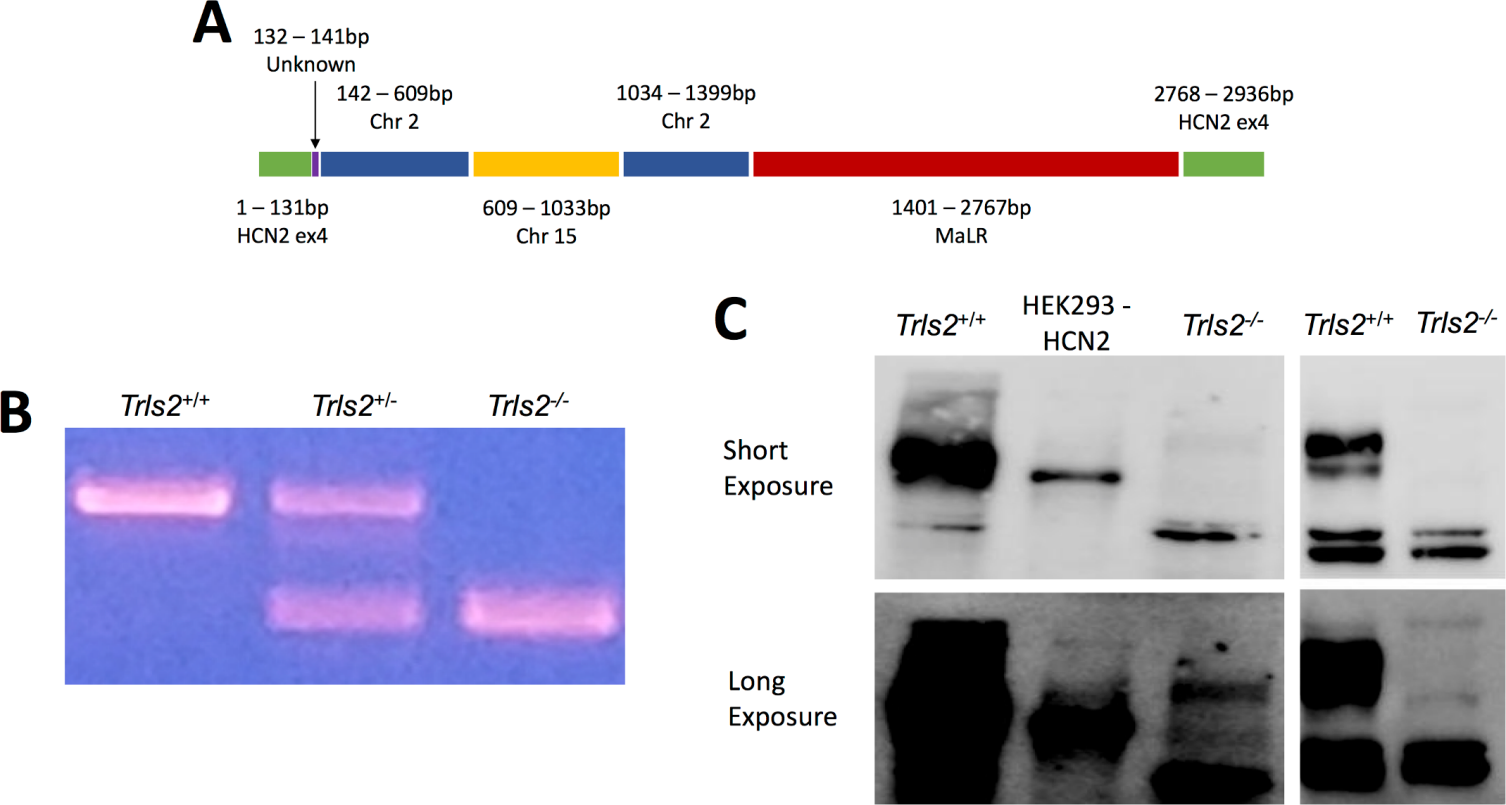
Insertion in exon 4 of *Hcn2* causes loss of protein in TRLS/2J mice. **A)** Sanger sequencing revealed a ~2.5kb insertion into exon 4 of HCN2. This mutation started with an unknown, 9bp sequence followed by genomic DNA from Chromosome (Chr) 2, genomic DNA from Chr 15, another distinct genomic sequence from Chr 2, and then a large Mammalian apparent LTR Retrotransposon (MaLR) sequence. **B)** Primers were made to genotype TRLS/2J mice based on the mutation sequence and could detect the wildtype allele (473bp) and the TRLS/2J allele (289bp). An example of this genotyping is shown from *Trls2*^+/+^, *Trls2*^+/-^, and *Trls2*^-/-^ mice. **C)** N-terminal (left panels) and C-terminal (right panel) antibodies against HCN2 revealed complete loss of protein in *Trls2*^-/-^ mice. The upper panels were taken with a short exposure while the lower panels were taken with a longer exposure to confirm that no protein band was present. Two non-specific bands are located in both *Trls2*^+/+^ and *Trls2*^-/-^ mice.

TRLS/2J mice begin to develop a tremor and wasting between the second and third week of life. TRLS/2J mice displayed non-complementarity when bred with a distinct mouse strain lacking HCN2 expression (BKS(Cg)-trls/J), suggesting that a mutation affecting *Hcn2* was present, and the affected allele was indeed mapped to chromosome 10[13]. To characterize the mutation leading to this phenotype, we sequenced *Hcn2* and found a 3kb insertion in the coding sequence of exon 4 (**Fig 1A**). This sequence is composed of genomic pieces of chromosomes 2 and 15 bookended by a small (9 bp) unidentified stretch of DNA on one side and unidentified DNA with many sequences similar to Mammalian apparent LTR Retrotransposons (MaLR) on the other. Based on this sequence information, we developed primers to genotype the mice (**Fig 1B**), and detection of the homozygous insertion allele always corresponded to the expected TRLS/2J phenotype. To determine the impact of this insertion, we performed western blots from hippocampal tissue with antibodies against the C- and N-terminus of HCN2, which confirmed a complete loss of protein (**Fig 1C**). These data suggest that the detected insertion in exon 4 of HCN2 is responsible for the TRLS/2J phenotype and results in loss of HCN2 protein.

### TRLS/2J mice display similar phenotypes to other mice lacking HCN2 expression

**Fig 2:**
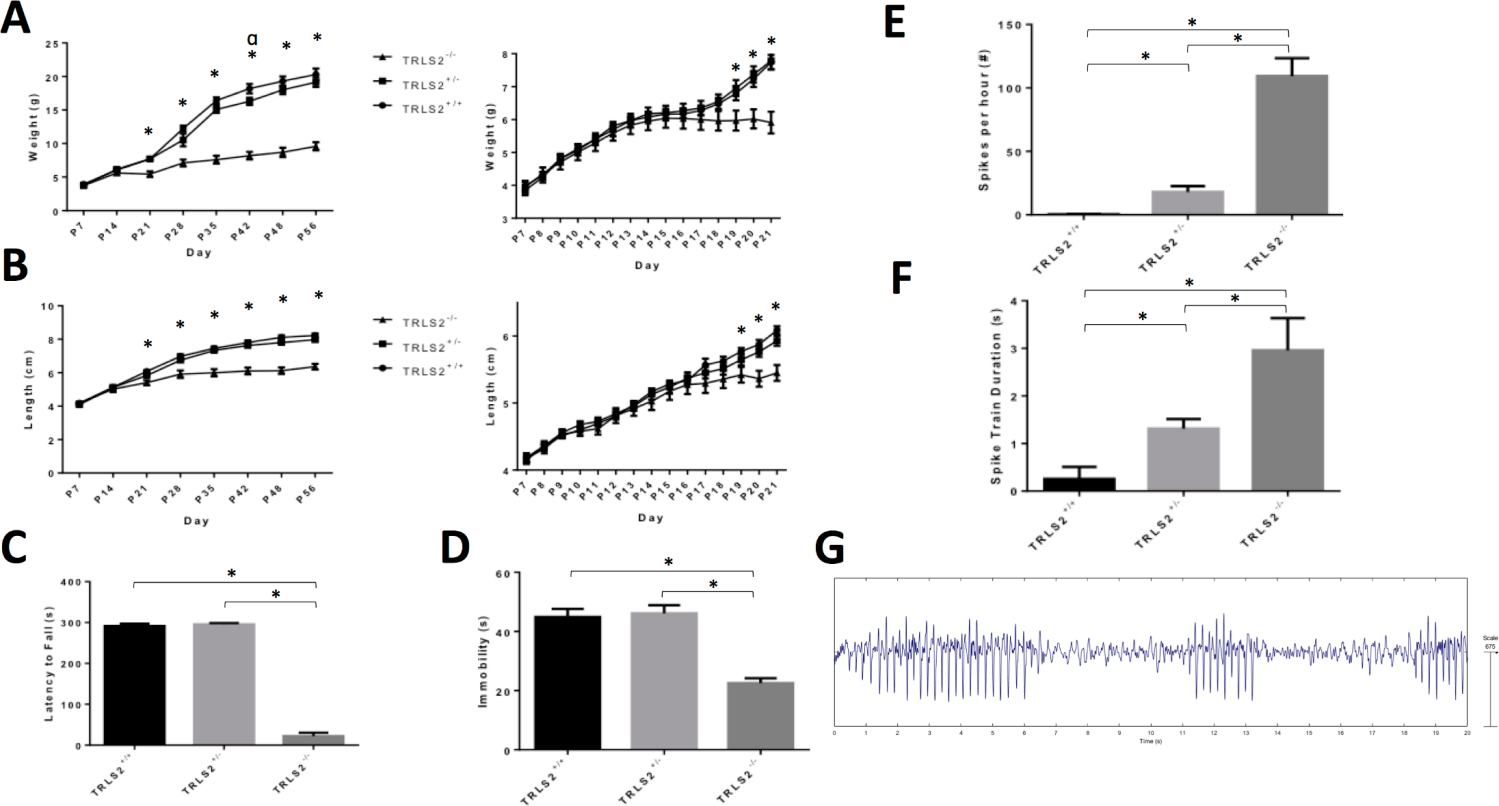
TRLS/2J mice show similar phenotypes to other mutant mice lacking HCN2 expression. **A)** Weights and **B)** lengths of *Trls2*^+/+^, *Trls2*^*+/-*^, and *Trls2*^-/-^ mice were obtained daily from P7 to P21 (right graphs) and weekly until P56 thereafter (left panel). *Trls2*^-/-^ mice began to fall of the growth curve between P14 and P21. (Repeated Measures Two-Way ANOVA. Weekly Weight: F_Time_(7,196) = 385.9, p < 0.0001; F_Genotype_(2,28) = 65.39, p < 0.0001, F_Interaction_(14,196) = 33.42, p < 0.0001. Weekly Length: F_Time_(7,196) = 676.9, p < 0.0001; F_Genotype_(2,28) = 36.58, p < 0.0001; F_Interaction_(14,196) = 27.09, p < 0.0001. Pre-weaning Weight: F_Time_(14,532) = 352.7, p < 0.0001; F_Interaction_(28,532) = 12.75, p < 0.0001. Pre-weaning Length: F_Time_(14,560) = 468.5, p < 0.0001; F_Interaction_(28,560) = 5.815). Tukey’s *Post Hoc* Test was used to detect individual differences between groups. An * denotes p < 0.05 between *Trls2*^+/+^ and *Trls2*^-/-^ as well as between TRLS2^+/-^ and TRLS2^-/-^. An α denotes p < 0.05 between *Trls2*^+/+^ and *Trls2*^+/-^. **C)** *Trls2*^-/-^ mice showed significantly shorter latencies to fall on the RotaRod (One-Way ANOVA. F(2,29) = 543.4, p < 0.0001; Tukey’s *Post Hoc* Test p < 0.05). **D)** *Trls2*^-/-^ mice showed significantly reduced immobility on the Tail Suspension Test (One-Way ANOVA. F(2,29) = 12.46, p < 0.0001; Tukey’s Post Hoc Test p < 0.05). **E)** During a 24hr monitoring period, one-way ANOVA revealed differences in the frequency (F(2,28) = 140.1, p < 0.0001) and **F)** durations (F(2.28) = 18.68, p < 0.0001) of Spike-Wave Discharges (SWDs) by genotype, and *Trls2*^-/-^ mice showed persistent SWDs while *Trls2*^+/-^ had prominent but fewer SWDs. Tukey’s *Post Hoc* Tests demonstrated that all groups exhibited different frequencies and durations of SWDs (p < 0.05). **G)** Representative 20s epoch of EEG from Trls2^-/-^ mouse. Significance (p < 0.05) is denoted with an *, and error bars represent Standard Error of the Mean. (n_+/+_ = 10–13, n_+/-_ = 12–19, n_-/-_ = 5–10).

Weight and length was recorded daily from P7 until P21 and then weekly until P56 thereafter for *Trls2*^+/+^, *Trls2*^+/-^, and *Trls2*^*-/-*^ mice. Beginning in the third week of life, the growth rate slowed in *Trls2*^*-/-*^ mice, leading to much lower weight and length measurements than their *Trls2*^+/+^ or *Trls2*^+/-^ siblings (**Fig 2A and 2B**). A significant tremor and difficulty with ambulation in the homecage was observed in *Trls2*^-/-^ mice during this time period. Though the *Trls2*^*-/-*^ mice were observed to eat hard chow, these mice showed early mortality shortly after weaning if diet gel was not supplemented. In addition, *Trls2*^*-/-*^ mice that had diet gel removed from their cage weeks to months after weaning also tended to die spontaneously within about a week of gel removal. For this reason, all mice in this study were given supplemental diet gel upon weaning, and many *Trls2*^*-/-*^ mice lived well beyond 12 months on this diet. However, even on this supplemental diet or in mice that were maintained on ground and moistened chow, the significant difference in size between *Trls2*^*-/-*^ mice and their siblings was readily apparent. *Trls2*^*+/-*^ mice were indistinguishable from their *Trls2*^+/+^ siblings.

Starting at 8 weeks of life, age- and sex-matched *Trls2*^+/+^, *Trls2*^+/-^, and *Trls2*^*-/-*^ mice were assessed for locomotor activity via RotaRod, antidepressant-like activity on the TST, and spike-wave discharges during 24hr video-EEG monitoring to determine if the *Trls2*^*-/-*^ mouse recapitulated the phenotype of previously described mice lacking HCN2 expression. Similar to *Hcn2* knockout and *apathetic* (*Hcn2*^*ap/ap*^) mutant mice[5, 6], *Trls2*^*-/-*^ mice showed a significantly decreased latency to fall from a rotating rod (**Fig 2C**). In addition, *Trls2*^*-/-*^ mice spent decreased time immobile on the TST (**Fig 2D**), consistent with an increase in antidepressant-like behavior. *Trls2*^+/+^ and *Trls2*^+/-^ mice performed similarly on both tests. This lack of immobility in *Trls2*^*-/-*^ mice was not due to their natural tremor, as only clear movements of the hindlimbs and forelimbs were counted as mobility. *Trls2*^*-/-*^ mice showed difficulty ambulating. When tested on a moving belt in the DigiGait apparatus, *Trls2*^*-/-*^ mice were unable to ambulate faster than 3cm/s, while *Trls2*^+/+^ and *Trls2*^+/-^ ambulated easily at over 24cm/s (data not shown). *Trls2*^+/+^ and *Trls2*^+/-^ mice performed similarly on both tests.

TRLS/2J mice were affixed with an EEG headset and monitored for 24 hours for detection of epileptiform activity. Consistent with other mice lacking HCN2 expression, *Trls2*^*-/-*^ mice showed persistent spike-wave discharges consistent with an absence epilepsy-like phenotype (**Fig 2E–2G**). In addition, prominent spike-wave discharges (SWDs) often coincided with behavioral freezing. Interestingly, *Trls2*^+/-^ mice showed some epileptiform activity as well, though the frequency and duration of these SWDs was reduced compared to *Trls2*^*-/-*^ mice. *Trls2*^+/+^ mice had nearly no SWDs. Thus, *Trls2*^*-/-*^ mice show similar deficits to other mice lacking HCN2 expression, further adding evidence that HCN2 influences multiple phenotypes.

### TRLS/2J mice display metabolic dysfunction consistent with nutritional deficiency

**Fig 3:**
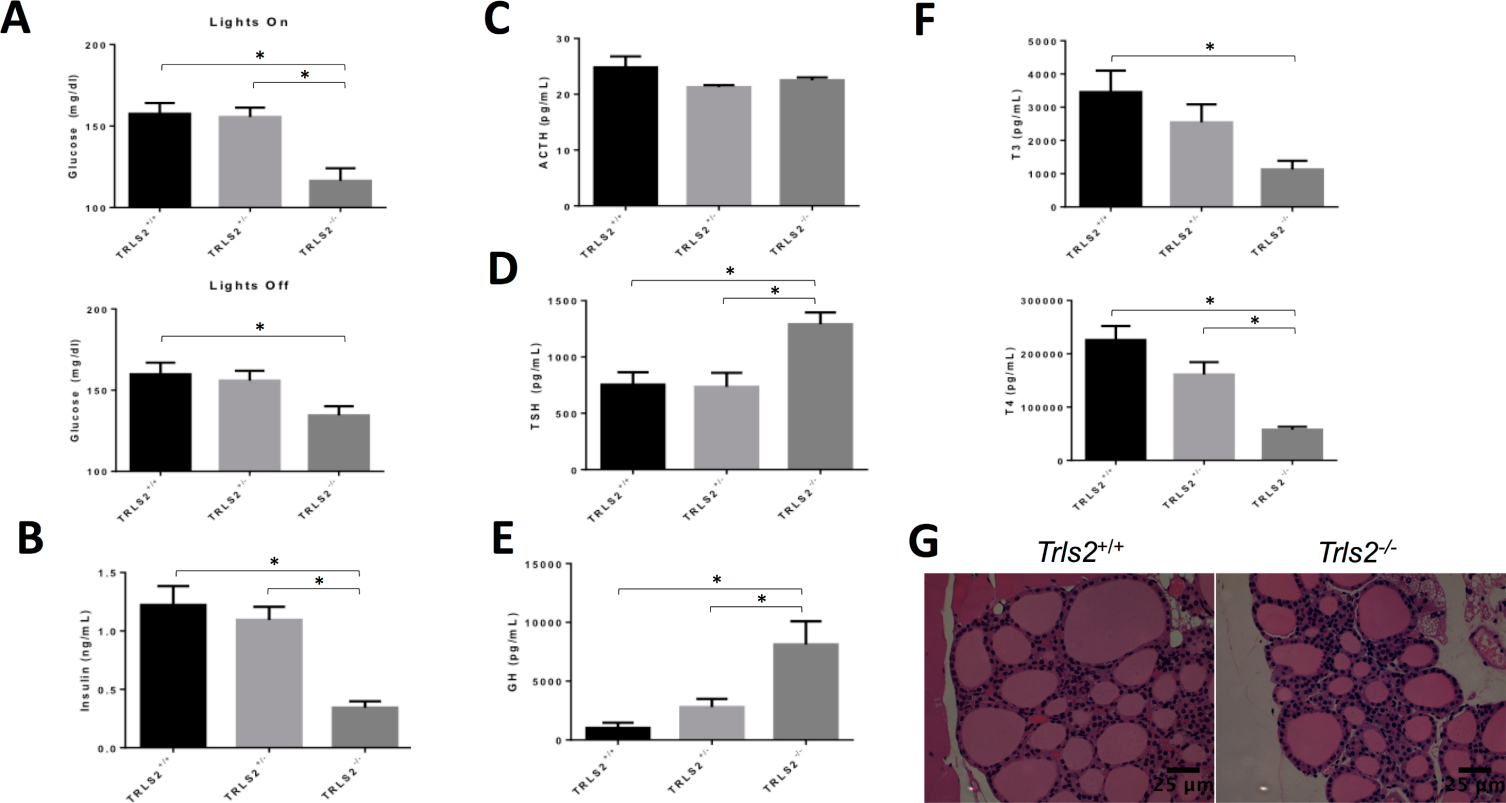
TRLS2^-/-^ mice demonstrate appropriate hormone responses to low blood glucose. **A)** Blood glucose was assessed at two time points in *Trls2*^+/+^, *Trls2*^+/-^, and *Trls2*^-/-^ mice, one hour after lights on (top graph) and an hour after lights off (bottom graph). *Trls2*^-/-^ mice show significantly lower blood glucose at both time points (One-Way ANOVA. Lights On: F(2,33) = 11.79, p < 0.0001. Lights Off: F(2,33) = 4.626, p < 0.05. Tukey’s *Post Hoc* Test p < 0.05). **B)** *Trls2*^-/-^ mice have significantly less serum insulin at one hour after lights off (One-Way ANOVA. F(2,30) = 13.72, p < 0.0001. Tukey’s *Post Hoc* Test p < 0.05). **C)** Serum ACTH levels are similar between *Trls2*^+/+^, *Trls2*^+/-^, and *Trls2*^-/-^ mice. **D)** Serum TSH (One-Way ANOVA. F(2,33) = 7.739, p < 0.01. Tukey’s *Post Hoc* Test p < 0.05). and **E)** GH (One-Way ANOVA. F(2,31) = 9.103, p < 0.001. Tukey’s *Post Hoc* Test p < 0.05) are increased *Trls2*^-/-^ mice. **F)** T3 (top graph; One-Way ANOVA. F(2,28) = 5.699, p < 0.01. Tukey’s *Post Hoc* Test p < 0.05) and T4 (bottom graph; One-Way ANOVA. F(2,32) = 17.96, p < 0.0001. Tukey’s *Post Hoc* Test p < 0.05) are decreased in *Trls2*^-/-^ mice. **G)** H&E sections (40x) of thyroid gland morphology is similar between *Trls2*^+/+^ and *Trls2*^-/-^, without signs of swollen follicles or dysplastic epithelial cells. Significance (p < 0.05) is denoted with an *, and error bars represent Standard Error of the Mean. (n_+/+_ = 10–12, n_+/-_ = 10–12, n_-/-_ = 10–12).

HCN2 expression has been reported in many neural and non-neural organs, including the brain, spinal cord, peripheral nerves, heart, pituitary gland, pancreas, GI tract, and kidneys. Of these tissues, many could influence the small size of these mice. To investigate what might cause the severe growth restriction in these mice, we assessed their non-fasting blood glucose levels at one hour after lights were turned on and one hour after they turned off. Though *Trls2*^+/+^ and *Trls2*^+/-^ mice showed similar glucose levels, *Trls2*^*-/-*^ mice had reduced blood glucose concentrations (**Fig 3A**).

Because HCN2 has been shown to impact beta-cell function in the pancreas[14], we measured the blood insulin levels of TLRS/2J mice at one hour after lights off. The low blood sugar at both time points suggested that a diabetic phenotype was unlikely. By contrast, if hyperinsulinemia was the cause of the restricted growth in these mice, we would expect higher than normal levels of serum insulin at this time point. However, while *Trls2*^+/+^ and *Trls2*^+/-^ mice displayed similar insulin levels, *Trls2*^*-/-*^ mice showed significantly decreased insulin (**Fig 3B**), which was expected given the hypoglycemia. Both the hypoglycemia and low insulin would be consistent with diminished food intake, which is examined in more detail below.

HCN2 is also expressed in the hypothalamus and pituitary gland, where HCN2 is broadly expressed in hormone-secreting cells[15] and has been shown to regulate hormone release in certain cell types[16]. Thus, we investigated whether pituitary hypofunction explained the growth restriction seen in TRLS/2J mice. Through assaying serum levels of pituitary hormones, we found that while ACTH levels were similar between *Trls2*^+/+^ and *Trls2*^-/-^ mice (**Fig 3C**), both TSH and GH were elevated in *Trls2*^-/-^ mice (**Fig 3D and 3E**). The increased GH is consistent with malnutrition[17], although TSH is generally low to normal in malnutrition[18]. An elevation in both these hormones is inconsistent with hypopituitarism due to loss of HCN2 expression.

The elevation in TSH led us to assay serum T3 and T4 levels. Compared to *Trls2*^+/+^ mice, *Trls2*^-/-^ mice showed decreased T3 and T4 (**Fig 3F**), suggesting an appropriate elevation of TSH in response to these low levels. Since potential explanations for these low thyroid levels could include either nutritional deficiency or congenital hypothyroidism due to gland dysfunction, we performed H&E stains of *Trls2*^+/+^ and *Trls2*^-/-^ thyroids. In transgenic models of congenital hypothyroidism in mice, TSH is generally high and the thyroid gland morphology is greatly altered[19–22]. In contrast to these cases, *Trls2*^+/+^ and Trls2^-/-^ mice had normal thyroid morphology without swollen colloidal cells, epithelial dysplasia, or hypoplastic follicles, suggesting appropriately active thyroid function (**Fig 3G**). In total, these results are more consistent with a nutritional deficiency underlying the growth restriction in *Trls2*^-/-^ mice rather than hormonal pancreas, pituitary gland, or thyroid dysfunction.

### TRLS/2J mice show decreased feeding and slow gut motility

**Fig 4:**
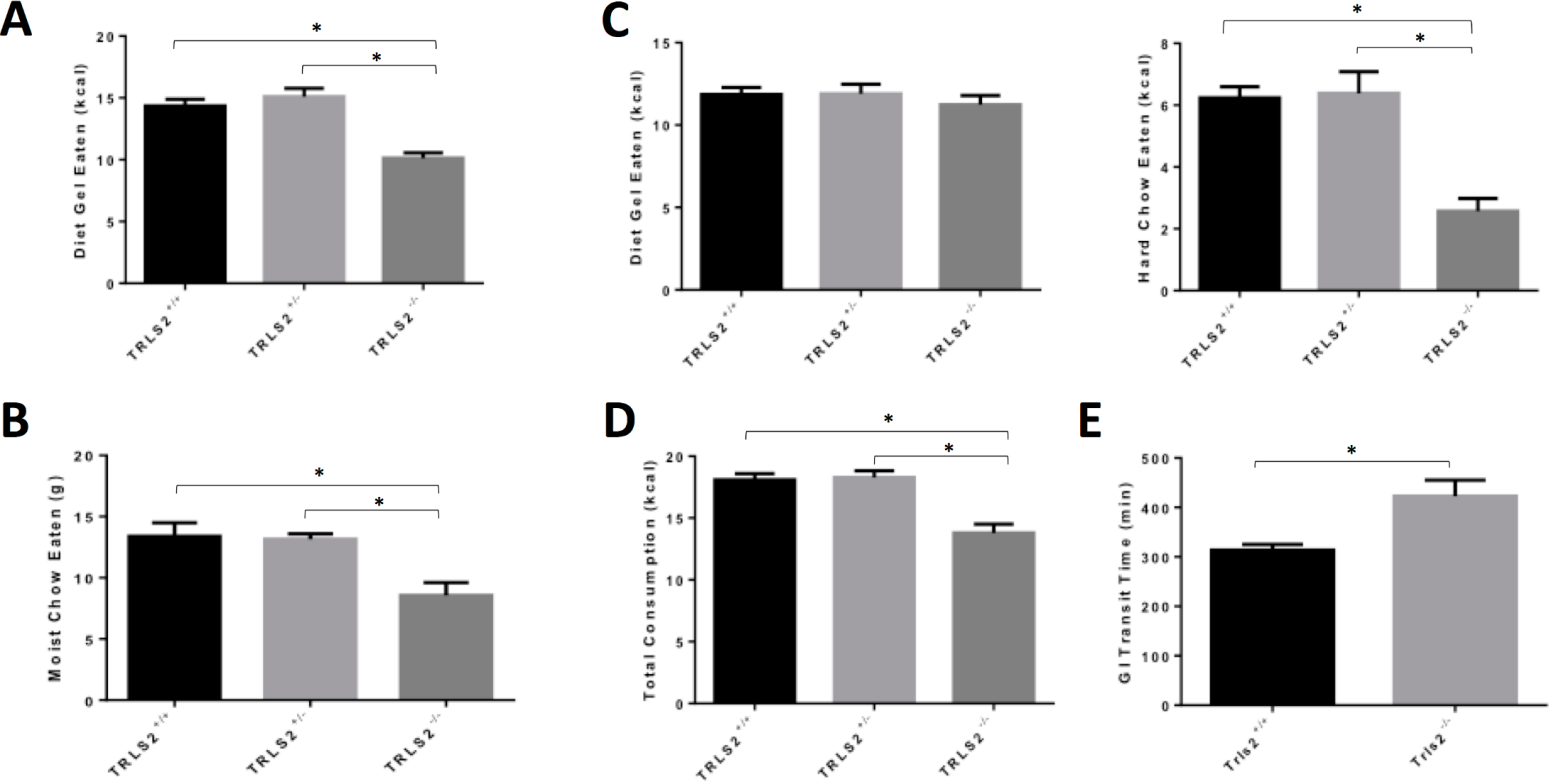
TRLS2^-/-^ mice feed less and have slow GI tract motility. **A)** *Trls2*^+/+^, *Trls2*^+/-^, and *Trls2*^-/-^ mice were given diet gel and water, and the amount of diet gel eaten was tracked during three consecutive 24 hour periods. The average amount of diet gel eaten by *Trls2*^-/-^ mice was significantly less than *Trls2*^+/+^ and *Trls2*^+/-^ mice (One-Way ANOVA. F(2,28) = 22.61, p < 0.0001. Tukey’s *Post Hoc* Test p < 0.05). **B)** *Trls2*^-/-^ mice eat less moist chow (One-Way ANOVA. F(2,20) = 8.749, p < 0.01. Tukey’s *Post Hoc* Test p < 0.05). **C)** When given a choice between diet gel and intact chow, Trls2^-/-^ mice eat similar amounts of diet gel to *Trls2*^+/+^ and *Trls2*^+/-^ mice but eat significantly less hard chow (One-Way ANOVA. F(2,21) = 16.93, p < 0.0001. Tukey’s *Post Hoc* Test p < 0.05). **D)** The total kcal consumed was calculated by adding the amount of hard chow and diet gel eaten, and Trls2^-/-^ mice ate significantly less than Trls2^+/+^ and Trls2^+/-^ mice (One-Way ANOVA. F(2,27) = 18.20, p < 0.0001. Tukey’s *Post Hoc* Test p < 0.05). **E)** Mice were gavaged with a red methylcellulose gel and the time for red feces to appear was noted for each mouse. *Trls2*^-/-^ had much slower transit times than *Trls2*^+/+^ mice (Student’s T-Test. t_23_ = 3.631, p < 0.01). Significance (p < 0.05) is denoted with an *, and error bars represent Standard Error of the Mean. (n_+/+_ = 9–15, n_+/-_ = 8–13, n_-/-_ = 7–10).

As mentioned above, *Trls2*^-/-^ mice often die before 4 weeks of age if they are not supplemented with diet gel. To investigate the feeding of these mice more rigorously, we tracked the amount of food eaten with three different diets over three weeks. First, we fed *Trls2*^+/+^, *Trls2*^+/-^, and *Trls2*^-/-^ mice diet gel alone for 3 days and measured the amount eaten over each 24-hour period. The following week, we fed the same cohort of mice moistened and ground chow. In the final week, mice were given a choice between diet gel and intact mouse chow. *Trls2*^+/+^ and *Trls2*^+/-^ mice ate similar amounts of all three diets, while *Trls2*^-/-^ mice ate significantly less diet gel and moist chow (**Fig 4A and 4B**). During the choice diet, *Trls2*^-/-^ mice ate similar amounts of diet gel to the *Trls2*^+/+^ and *Trls2*^+/-^ mice, but they ate significantly less of the normal chow and consumed less calories overall (**Fig 4C and 4D**). As all three diets were placed on the floor of the mouse’s cage, difficulty in ambulation was unlikely to be the cause of the reduction in the amount of food eaten.

The *Trls2*^*-/-*^ mice show poor growth starting during the second and third week of life before weaning. Because the gastrointestinal (GI) tract continues to develop in mice during this time[23, 24], we next investigated whether *Trls2*^-/-^ mice had appropriately developed GI structures by studying the morphology of the adult *Trls2*^-/-^ GI tract. Though all the GI structures in *Trls2*^-/-^ mice were observed to be smaller than *Trls2*^+/+^ mice, and the lengths of the small and large intestines were significantly shorter **(S1 Fig)**, there were no major morphological anomalies identified in the stomach, duodenum, jejunum, ileum, cecum, or colon (**S2–S4 Figs**). The small size of these organs was consistent with the overall smaller size of these mice in general, and similarly smaller structures were noted in the skin, which was grossly thinner than for *Trls2*^+/+^ mice and was reduced in size on H&E (**S5 Fig**).

As reduced food consumption can be a symptom of GI dysmotility, we next assessed GI Transit Time (GTT). After fasting *Trls2*^+/+^ and *Trls2*^-/-^ mice for 18 hours with free access to water, we gavaged mice with a 3% methylcellulose plus carmine red solution and measured the amount of time before the first red feces appeared. Consistent with a dysmotility phenotype, *Trls2*^-/-^ mice showed significantly slower GTT (**Fig 4D**). This change in GTT was observed without major differences in stool consistency between *Trls2*^+/+^ and *Trls2*^-/-^ mice, suggesting that slow gut motility may lead to reduced energy intake in *Trls2*^-/-^ mice, potentially playing a role in their observed growth restriction.

## Discussion

In this report, we have identified a pathologic mutation in *Hcn2* in the TRLS/2J mouse line and demonstrated that the phenotype of this mouse is similar to other mice with mutations leading to loss of HCN2 expression. Through investigation of insulin, pituitary hormones, and thyroid hormones, we present evidence that nutritional deficiency due to reduced energy intake rather than primary pancreatic, pituitary, or thyroid dysfunction causes the small size in *Trls2*^-/-^ mice. Finally, we show that altered GI motility is a plausible explanation for the reduced feeding and growth deficits in these mice.

### Growth restriction in TRLS/2J mice is unlikely to be due to hormonal dysfunction

Though HCN channels are generally studied in the heart and brain, these channels have been found to be expressed and regulate function in multiple organ systems [14–16, 25–33]. In particular, HCN2 has been reported to have a functional role in many hormone secreting tissues, including the pancreas and pituitary[14, 16]. In the β-cells of the pancreas, overexpression of HCN2 increased insulin secretion in response to glucose, though a dominant negative isoform of HCN only reduced insulin secretion during hypokalemic conditions by a very small amount[14]. In the pituitary, HCN2 is expressed in many pituitary cells, and I_h_ is detectable in gonadotrophs, thyrotrophs, somatotrophs, lactotrophs, and other unidentified cell types [15]. The functional impact of HCN2 was characterized in cultured lactotrophs, where HCN2 regulates hormone exocytosis[16]. However, our quantification of growth-related hormones from these tissues was more consistent with malnutrition than dysfunction in hormone release.

The elevated TSH and decreased thyroid hormones are qualitatively consistent with congenital hypothyroidism. However, mice with congenital hypothyroidism are small from birth, and the difference in size is usually apparent much earlier than when we saw *Trls2*^*-/-*^ mice fall off the growth curve[19, 20, 22]. In addition, T3 and T4 levels were not completely absent and TSH was appropriately elevated, suggesting regulation along the pituitary-thyroid axis was intact. Finally, congenital hypothyroidism almost always leads to noticeable histopathology [19–22], which was not found in Trls2^-/-^ mice. Thus, thyroid dysfunction is unlikely to explain growth restriction in these mice.

Though we cannot exclude more subtle changes in hormone timing, such as changes in the pulsatile release of growth hormone, we believe that these more subtle changes would be unlikely to lead to the severe growth restriction observed in these mice, which is much less severe in mice with specific deficiencies in pulsatile release[34]. In addition, while we did not assay cortisol directly, the similar levels of ACTH in Trls2^+/+^ and *Trls2*^*-/-*^ mice suggests normal HPA axis function. In summary, we believe that the growth restriction observed in mice lacking HCN2 expression is unlikely to be due to primary dysfunction in hormone secretion from the pancreas, pituitary, or thyroid.

### Growth restriction in TRLS/2J mice is likely due to malnutrition secondary to GI dysmotility

The low glucose, mixed hormonal panel, and timing of growth restriction suggest malnutrition as the cause of the poor growth in TRLS/2J mice. During the second to third week of life, when the growth restriction appears, two important events occur: mice start eating solid food in addition to mother’s milk, and the intestines mature and form villi[23, 24]. Thus, difficulties in feeding, digestion, or lack of normal GI development could lead to the malnourished state of these mice. Based on the reduced feeding observed in Trls2^-/-^ mice and normal GI histology, our results suggested decreased energy intake to be the most likely cause. In addition, it was interesting to note that *Trls2*^-/-^ mice eat proportionately less than their *Trls2*^+/+^ and *Trls2*^+/-^ littermates as the diet becomes more fibrous, as *Trls2*^-/-^ mice eat only slightly less diet gel but much less hard chow by weight or kcal than their *Trls2*^+/+^ and *Trls2*^+/-^ littermates. However, because *Trsl2*^-/-^ mice clearly ate all the food types and were observed to eat hard chow without difficulty, we believe it is unlikely that the growth restriction was due to ambulatory dysfunction, voluntary motor deficits, or food palatability. Instead, we observed that *Trls2*^-/-^ mice had reduced GTT compared to *Trls2*^+/+^ littermates, and because stool consistency was generally similar between all TRLS/2J mice, differences in GI motility rather than fluid secretion in the GI tract seemed more likely. When combined with the observation that *Trls2*^-/-^ mice had a harder time eating solid food, these data suggest that reduced energy intake secondary to GI dysmotility may cause the growth restriction observed in these mice.

Precedence for slow GTT associating with impaired growth and early mortality has been documented before in transgenic mice, namely the Guanylyl Cyclase Knockout (GC-KO) mouse [35]. This mouse showed many similarities to the TRLS/2J mouse, including early mortality shortly after weaning, reduced body weight, dependence on a non-fibrous diet, and greatly impaired GTT. Though NO-cGMP has been shown to affect HCN2 activity [36], a direct link between the two in the GI tract has not been established.

The presence of HCN channels in the GI tract has been observed by many groups. For most studies, I_h_ or HCN2 was detected specifically in Enteric Nervous System (ENS) neurons[29–31], and one study reported that HCN2 was localized to ChAT+ cells but not ICCs in the mouse GI tract [37]. These ChAT+ cells are likely to be acetylcholine secreting, ENS neurons, which may affect gut motility both through direct innervation of smooth muscles and ICCs. In conjunction, HCN channels in ICCs are very likely to influence of the pacemaking ability of these neurons, as three different HCN blockers abolished pacemaking activity in cultured colonic ICCs. However, it is unclear if HCN2 is expressed endogenously in these cells in vivo [33]. Determining if HCN2 loss in these ChAT+ neurons leads to GI dysmotility and growth restriction would require further experiments. In addition, the ENS has been shown to influence enteroendocrine cells[38], and it is unknown if HCN2 may further influence metabolism or energy intake through local hormone release through ENS regulation of these cells.

Decreased gut motility can be an incredibly distressing symptom that manifests during the course of many diseases. While primary dysmotility disorders, such as Chronic Intestinal Pseudo-obstruction (CIP) and Hirchsprung’s diease[39], can lead to anorexia and weight loss, secondary gut dysmotility is common in numerous other diseases, including diabetes[40, 41], Parkinson’s Disease[42, 43], and severe illness resulting in ICU admittance[44]. The impact of GI motility in these diseases can be quite severe, as many patients with gasteroparesis lose >10% of their body weight and 40% of those with gasteroparesis are resistant to pharmacotherapy[45]. It is interesting to note that HCN channel expression has been shown to be altered in patients with Hirchsprung’s Disease[46] and rodent models of diabetes[47] and Parkinson’s Disease[48], though no firm association between HCN dysfunction and the gastric dysmotility observed in these diseases has yet been uncovered. Still, the evidence presented here suggests that HCN2 may have an important role in regulating GI motility, and future investigations of HCN2 in terms of GI motility may uncover new mechanisms for disease pathogenesis and hopefully new treatments for patients with symptoms of slowed GI motility.

## Supporting information

S1 Fig *Trls2^-/-^* mice have short GI tracts. Despite the increase in GI Transit Time, *Trls2*^*-*.*-*^ mice have significantly shorter small (t_4_ = 6.667, p < 0.01) and large (t_4_ = 6.874, p < 0.01) intestines than *Trls2*^*+/+*^ mice. Significance (p < 0.05) is denoted with an *, and error bars represent Standard Error of the Mean. (n = 3)
(PDF)

S2 Fig *Trls2*^+/+^ and *Trls2*^-/-^ mice have similar gastric morphology. **A)** H&E sections (10x top panels; 20x bottom panels) of glandular and **B)** distal stomach.
(PDF)

S3 Fig *Trls2*^+/+^ and *Trls2*^-/-^ mice have similar morphology of small intestines. H&E sections (10x top panels; 20x bottom panels) of duodenum, B) jejunum, and C) Ileum.
(PDF)

S4 Fig *Trls2*^+/+^ and *Trls2*^-/-^ mice have similar morphology of the large intestines. **A)** H&E sections (10x top panels; 20x bottom panels) of cecum and **B)** distal colon.
(PDF)

S5 Fig *Trls2*^+/+^ and *Trls2*^-/-^ mice have similar skin morphology. **A)** H&E sections (10x top panels; 20x bottom panels) of foot pads.
(PDF)

S1 File Raw data. Data used for statistics and to create figures.
(XLSX)
