# Supplementary figures and images for: Loss of HCN2 leads to delayed gastrointestinal motility and reduced energy intake in mice
Source: PLoS One. 2018 Feb 21;13(2):e0193012. doi: 10.1371/journal.pone.0193012 (PMC5821371; doi:10.1371/journal.pone.0193012)

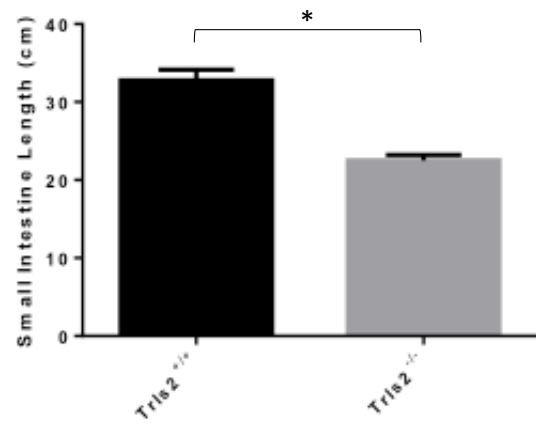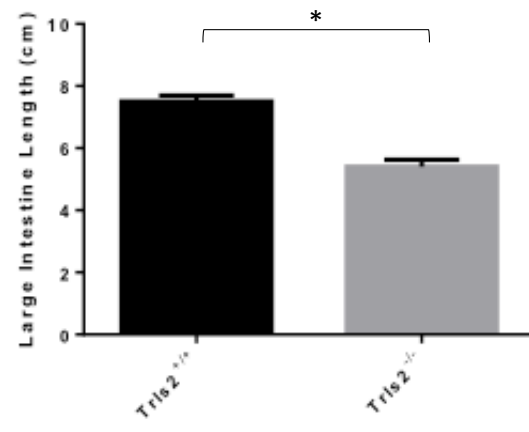

Supplement: S1 Fig — Despite the increase in GI Transit Time, Trls2-.- mice have significantly shorter small (t4 = 6.667, p < 0.01) and large (t4 = 6.874, p < 0.01) intestines than Trls2+/+ mice. Significance (p < 0.05) is denoted with an *, and error bars represent Standard Error of the Mean. (n = 3) (PDF) [file pone.0193012.s001.pdf]

**A**

*Trls2*<sup>+/+</sup>

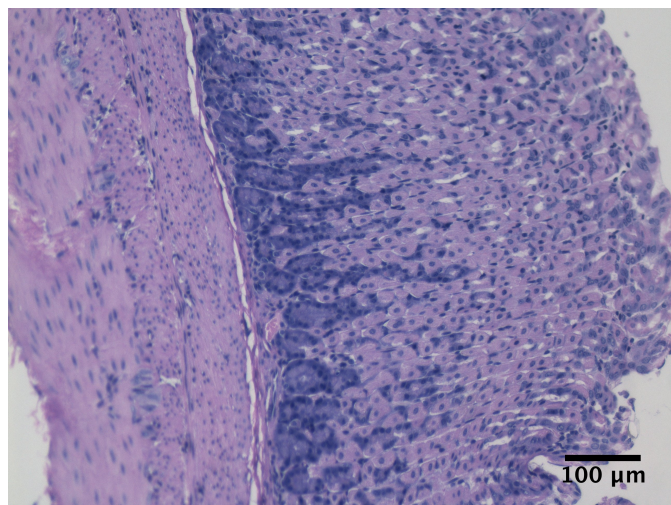

*Trls2*<sup>-/-</sup>

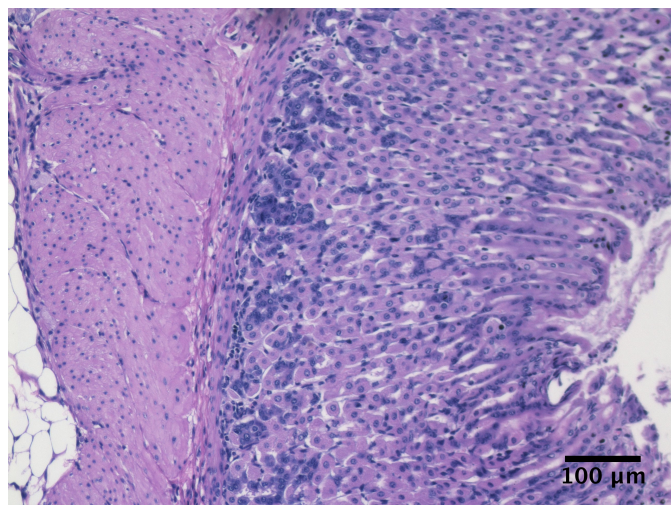

**B**

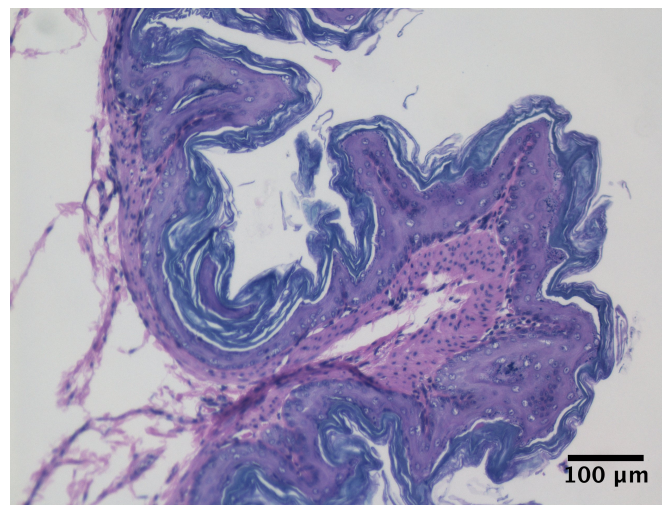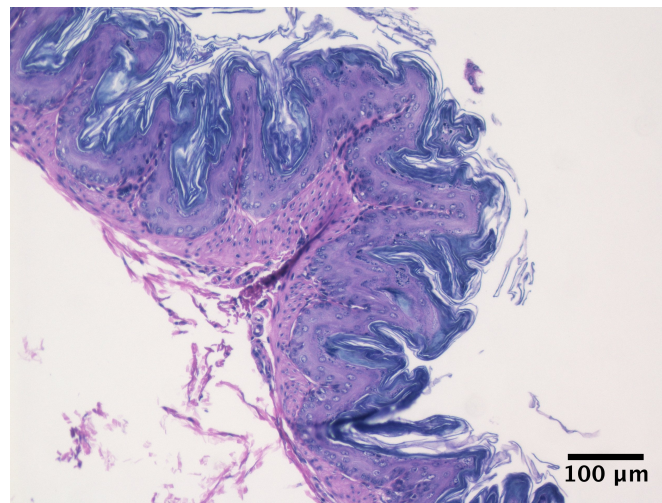

Supplement: S2 Fig — A) H&E sections (10x top panels; 20x bottom panels) of glandular and B) distal stomach. (PDF) [file pone.0193012.s002.pdf]

**A***Trls2*<sup>+/+</sup>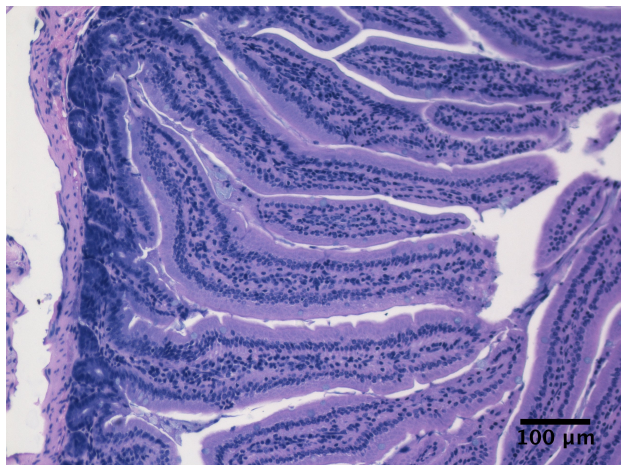**B**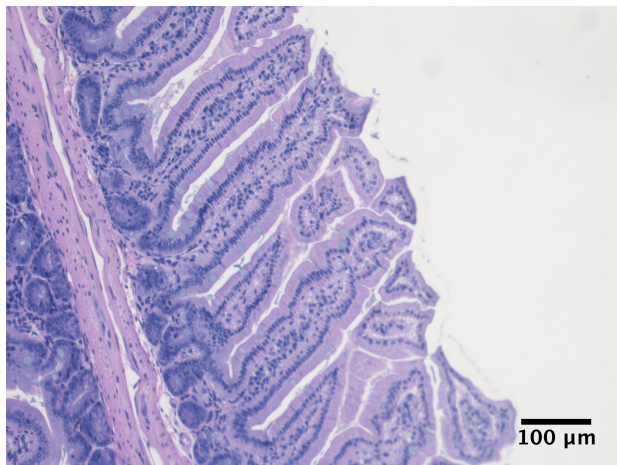**C**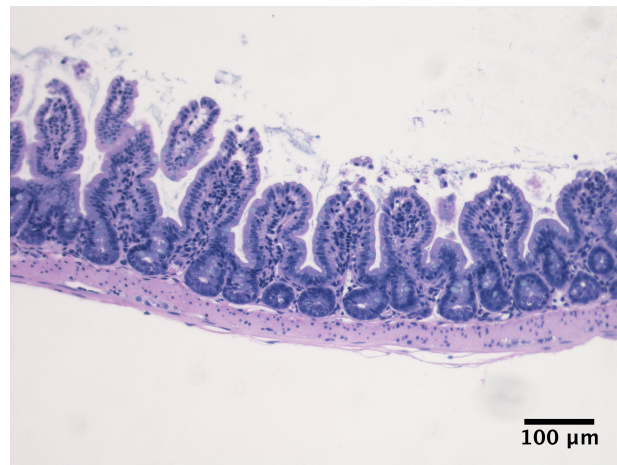*Trls2*<sup>-/-</sup>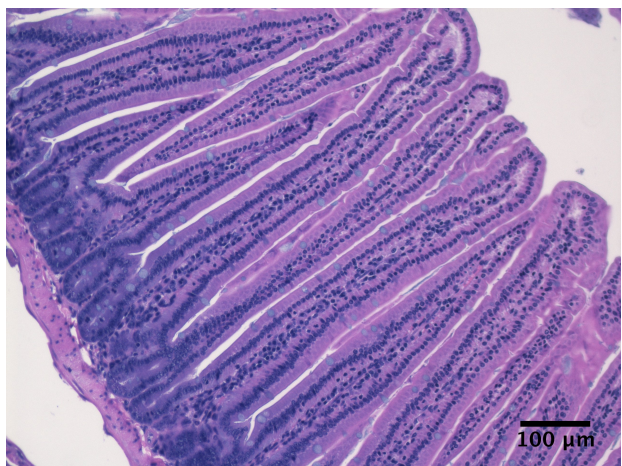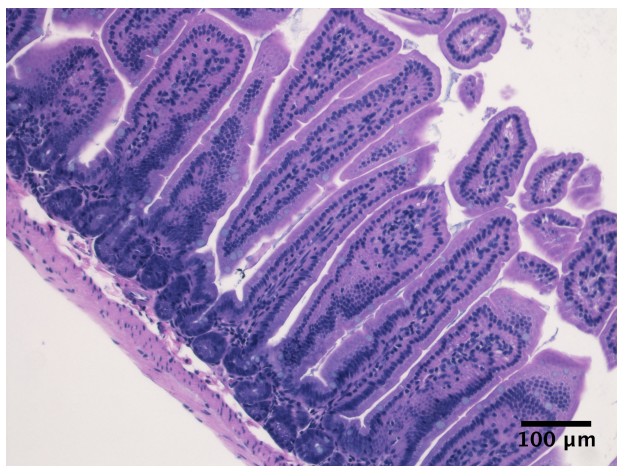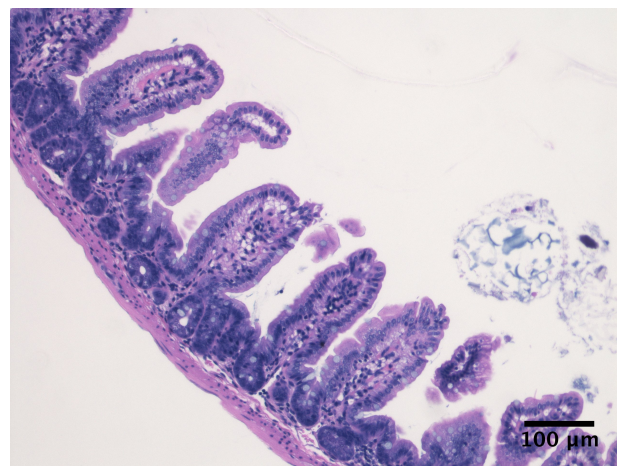

Supplement: S3 Fig — H&E sections (10x top panels; 20x bottom panels) of duodenum, B) jejunum, and C) Ileum. (PDF) [file pone.0193012.s003.pdf]

**A**

*Trls2*<sup>+/+</sup>

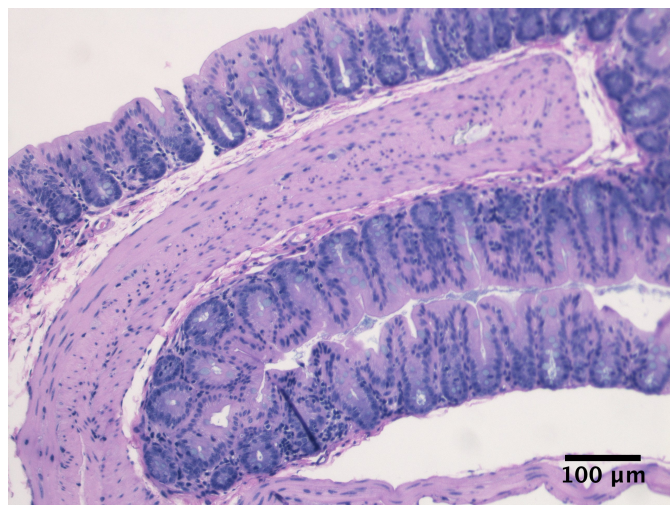

*Trls2*<sup>-/-</sup>

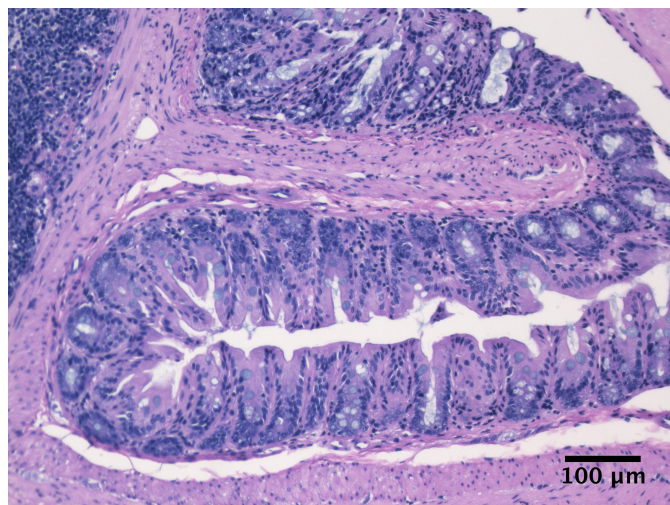

**B**

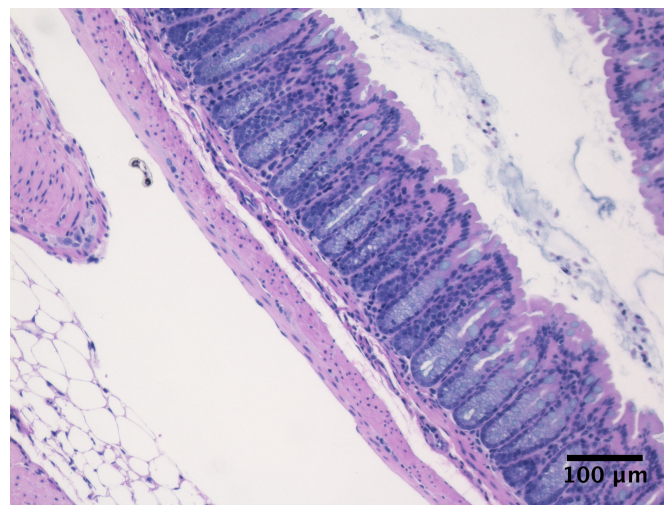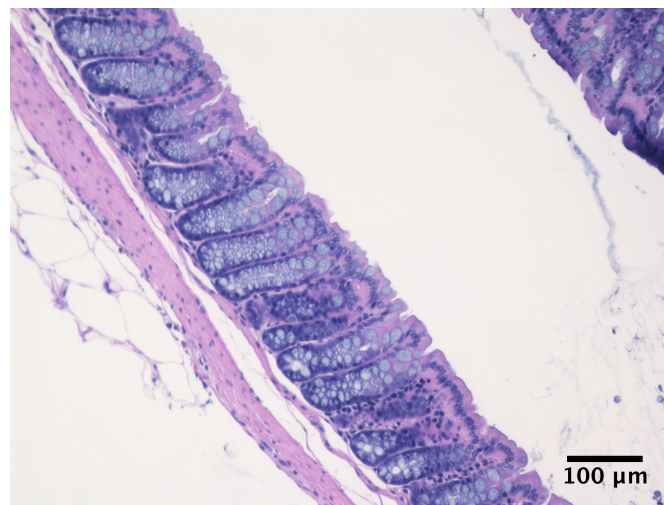

Supplement: S4 Fig — A) H&E sections (10x top panels; 20x bottom panels) of cecum and B) distal colon. (PDF) [file pone.0193012.s004.pdf]

*Trls2*<sup>+/+</sup>

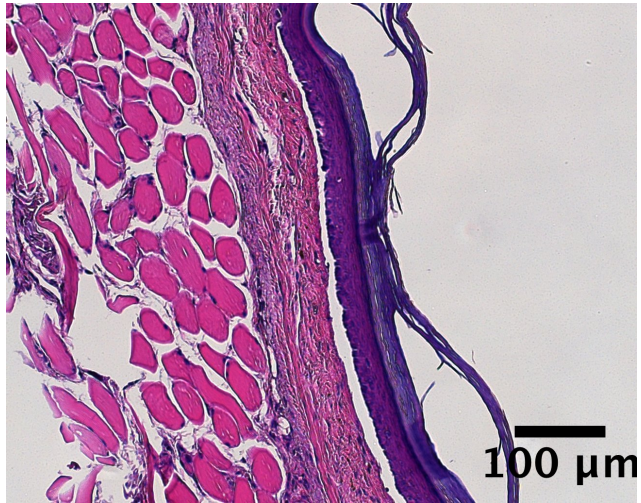

*Trls2*<sup>-/-</sup>

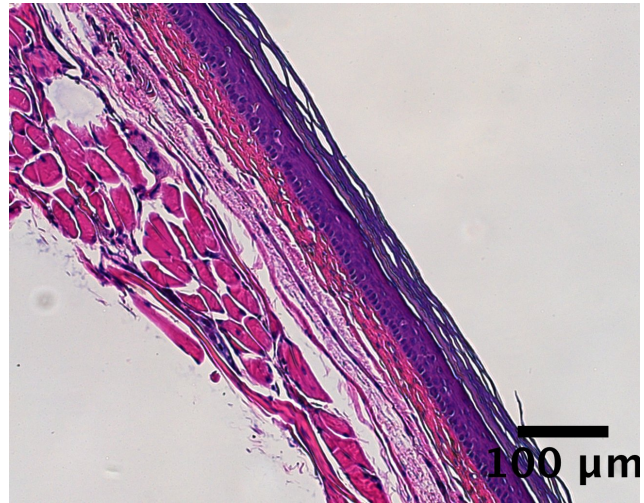

Supplement: S5 Fig — A) H&E sections (10x top panels; 20x bottom panels) of foot pads. (PDF) [file pone.0193012.s005.pdf]
